# Dietary Soluble Non-Starch Polysaccharide Level Influences Performance, Nutrient Utilisation and Disappearance of Non-Starch Polysaccharides in Broiler Chickens

**DOI:** 10.3390/ani12050547

**Published:** 2022-02-22

**Authors:** Hong T. Nguyen, Michael R. Bedford, Shu-Biao Wu, Natalie K. Morgan

**Affiliations:** 1 School of Environmental and Rural Science, University of New England, Armidale, NSW 2350, Australia; hnguye43@myune.edu.au (H.T.N.); swu3@une.edu.au (S.-B.W.); 2 AB Vista Woodstock Court, Blenheim Road, Marlborough SN8 4AN, UK; mike.bedford@abvista.com; 3 School of Molecular and Life Sciences, Curtin University, Bentley, WA 6102, Australia

**Keywords:** broiler, non-starch polysaccharide degradation, growth performance, nutrient digestibility

## Abstract

**Simple Summary:**

Non-starch polysaccharides (NSP) constitute a major part of the dietary fibre component in plant-based feed ingredients, accounting for approximately 10% of the nutrients in a poultry diet. However, NSP are generally not considered during formulation of commercial broiler diets. The functions of dietary NSP in poultry diets, including both the soluble and insoluble fraction, has been extensively researched and discussed. The soluble fraction is of particular interest to poultry nutritionists and producers, as it increases digesta viscosity, affecting nutrient digestion and absorption and thus litter quality. Soluble NSP (sNSP) also provides fuel for beneficial microbiota species. The extent of impact of dietary sNSP level on broiler performance and nutrient utilisation is poorly understood. Consequently, in this study, broilers were fed commercial-type diets with varying sNSP levels, and the effects of the sNSP level on ileal and total tract nutrient digestibility and productive performance were evaluated. The results revealed that even a small variation in dietary sNSP content induces an impact in broilers, particularly in young birds. Thus, sNSP level and composition should be considered during formulation of commercial poultry diets.

**Abstract:**

This study evaluated the effect of dietary soluble non-starch polysaccharides (sNSP) on performance and nutrient utilisation in broilers from d 0 to 35. Cobb 500 broilers (*n* = 480, 80 birds per treatment) were fed either wheat- or corn-soybean meal-based diets formulated to contain either a high, medium, or low sNSP content, in a 2 × 3 factorial arrangement, fed as Starter (d 0–14) and Grower (d 14–35). Birds fed the low sNSP level presented greater BWG at d 0–14 and lower feed intake at d 14–35 compared to birds fed the medium sNSP level (*p* < 0.005). At d 14, birds fed the high sNSP level presented greater ileal and total tract starch digestibility and total tract sNSP degradability and insoluble NSP degradability, compared to feeding the low sNSP level. At d 35, total tract DM and metabolisability of gross energy was greater in birds fed the medium sNSP level compared to those fed the high or low sNSP level (*p* < 0.005). Generally, bird performance and nutrient utilisation was greater in birds fed the corn-based diets compared to the wheat-based diets. These results illustrate that dietary sNSP level and composition influences bird performance and nutrient digestibility.

## 1. Introduction

The successful and sustainable development of broiler production, particularly during an era with reduced reliance on in-feed antibiotics and escalated ingredient prices, relies on minimising feed costs and managing bird gastrointestinal health. The majority of a poultry ration is derived from plant-based ingredients, which contain approximately 10–30% non-starch polysaccharides (NSP) [1]. Dietary NSP directly and indirectly affects nutritive value of the diet, as well as digestive function and metabolic processes [2], through its impact on development and morphology of the gastrointestinal tract [3,4] and on microbiota population and composition [5,6]. Consequently, there is increasing interest in the positive effects of feeding dietary NSP to poultry, including supplementing diets directly with sources of fibre [7,8]. However, crude, acid detergent, and neutral detergent fibre values are currently used as the indicator of dietary fibre availability for the bird, despite it being well-established that these values do not fully describe the actual fibre level or their physico-chemical properties in feedstuffs [1,9]. Total dietary fibre is defined as the sum of NSP and lignin [1,10]. This suggests that NSP values should be applied to poultry diet formulations in order to achieve the most from the fibre available in feed ingredients, and from NSP-degrading enzyme application.

The influence of dietary NSP in the gastrointestinal tract is dependent on its quantity and physio-chemical composition and structure [11,12]. Of particular interest is its solubility in water, with soluble and insoluble NSP presenting very different properties [13]. Commercial broiler diets contain around 10–12% total NSP, of which 1–2.5% is water soluble [14]. Soluble NSP (sNSP) with high molecular weight has a high water-holding capacity, resulting in increased viscosity and thus heightened water consumption, increased excreta moisture, and poorer litter quality, alongside increased endogenous secretions and thus nutritional losses [14,15,16,17]. In contrast, sNSP can also provide a source of fermentable substrates for beneficial intestinal bacteria through selective fermentation [18], resulting in production of short chain fatty acids, which provide a source of energy, and reduced ability for pathogenic bacteria to proliferate [19].

Generally, fibre content and composition is considered to be an issue only in diets containing viscous grains with high sNSP level, such as wheat and barley, which contain approximately 10–12% and 17–20% NSP, respectively (DM basis) [11]. Approximately 19% and 29% of the total NSP is water soluble in wheat and barley, respectively [20]. However, positive effects of supplementing NSP-degrading enzymes are also observed when feeding grains with low sNSP level, namely corn which has a total NSP content of approximately 8% (DM basis), less than 2% of which is soluble [21,22,23,24,25]. This suggests that the sNSP content should be accounted for when feeding both viscous and non-viscous grains, as even low levels of NSP can induce a negative impact on bird nutrient utilisation and performance. This was illustrated by Morgan et al. [15], who observed significant differences in feed conversion ratio and ileal digesta and excreta dry matter content when feeding diets with sNSP content ranging from 5.29–14.63 g/kg.

NSP-degrading enzymes are commonly applied to poultry diets, primarily as a tool to eliminate viscosity effects, but the sNSP substrates are not currently accounted for, which may partially explain the inconsistent results when applying these enzymes. This suggests that there is potential to manipulate dietary sNSP level to enhance gut health, and that it is beneficial to account for sNSP concentration and composition in feed ingredients when formulating diets. This was shown by Musigwa et al. [26], who reported an interaction between dietary sNSP to total NSP ratio and multi-carbohydrase supplementation on energy utilisation in broilers. This reaffirms the need to accurately quantify and account for NSP level and composition in diets fed to poultry. Currently, there is a deficit of data available about the levels of NSP to formulate to, and data that is available has been derived from studies of feeding diets specifically enriched with sources of NSP [27]. The NSP used in these diets is usually in purified form, which provides insight into the effects and mechanisms of NSP, but lacks industry relevance. Consequently, there is a lack of understanding about how sNSP level influences broiler performance in common commercial-type diets.

In this study, birds were fed commercial-type diets formulated to have similar crude fibre, energy, and protein contents but varying sNSP levels (low, medium, and high). The aim of this design was to both determine how much influence sNSP has on bird performance and nutrient utilisation and assess how reliable sNSP values are compared to crude fibre values during feed formulation. Wheat- and corn-based diets were used due to their differing NSP compositions, and to represent diets from different regions. The diets with ‘medium’ sNSP level represented the average sNSP level for commercial-type wheat- and corn- based diets in Australia, and the relative increase or decrease in sNSP level provided the ‘high’ and ‘low’ treatments, respectively. The hypothesis of this study was that sNSP level would have a direct impact on bird performance and nutrient utilisation, with a low level of sNSP having a detrimental impact, primarily due to lack of fuel for beneficial microbiota species. It was also hypothesised that outputs from this study would prove that crude fibre values are meaningless during feed formulation, and that NSP levels need to be considered.

## 2. Materials and Methods

### 2.1. Birds and Housing

Cobb 500-day-old mixed sex broilers (*n* = 480) were obtained from a local commercial hatchery at day of hatch. Upon arrival, birds were weighed and randomly distributed into 48 floor pens (120 cm length × 77 cm width), with ten birds per pen, bedded on clean wood shavings. The room was thermostatically controlled to produce an initial temperature of 32 °C, gradually reduced to 22 °C by d 21, and maintained until d 35. The lighting regimen used was 23 h of light at approximately 40 lux on d 1, with darkness increasing 1 h a day until 6 h of darkness was reached, and then 18 h of light per day at 10 lux was maintained for the remainder of the study. Feed and water were provided ab libitum. Starter diet was fed as crumble from d 0–7 and as pellets from d 8–14, and grower diet was fed as pellets from d 15–35. All experimental procedures were approved by the University of New England Animal Ethics Committee, Australia (Approval number: AEC18-058).

### 2.2. Diets and Experimental Design

All diets were formulated to meet or exceed the nutritional requirements for Cobb 500 broilers [28]. Prior to feed formulation, ingredients were ground through a 0.5 mm screen and the energy, protein, amino acid, mineral content, and crude fibre was analysed by near-infrared spectroscopy (NIRS, Evonik AminoProx, Essen, Germany). Soluble and insoluble NSP content was also analysed in the feed ingredients (Table 1) by measuring the constituent sugar components as alditol acetates using gas-liquid chromatography (Model CP3800, Varian Inc., Palo Alto, CA, USA), following the procedure of Englyst et al. [29] with some modifications as described by Theander et al. [30] and Morgan et al. [31]. Briefly, the sample was fat extracted using hexane and then free oligosaccharides were extracted by heating the sample at 80 °C with 80% ethanol. The starch in the resulting residue was gelatinised using acetate buffer (pH 5) and α-amylase and amyloglucosidase was added, at 95 °C and 55 °C, respectively, to remove the starch. The prepared sample was then incubated and centrifuged at 2000× *g* for 10 min and the resulting supernatant and residue were used for the analysis of soluble and insoluble NSP, respectively. For the soluble NSP analysis, the sugars released by the enzymes were removed using ethanol at 4 °C, the residue was dried and then 2 M trifluoroacetic acid added and heated at 125 °C. For the insoluble NSP analysis, the glucose released from starch digestion was removed with water and acetone, and the resulting supernatant was removed and the residue was dried. Following this, 12 M H_2_SO_4_ was added and the sample was heated to 35 °C, and then water was added and the sample was heated to 100 °C, cooled and then centrifuged at 3000× *g* for 15 min to sediment the insoluble materials. For the free sugar analysis, the extracted sample was dried, hydrolysed with 1 M H_2_SO_4_ at 100 °C, and centrifuged to sediment the insoluble material. Ammonium (28%) was added to an aliquot of the resulting supernatant from the insoluble NSP and free oligosaccharide samples. For all the resulting samples, an internal standard was added (allose, 4 mg/mL) and the sample was evaporated to dryness, and then re-dissolved in water with slight alkalinity. Freshly prepared NaBH_4_ was then added, the sample was incubated, and any excess NaBH_4_ was decomposed with glacial acetic acid. 1-methylimidazole and acetic anhydride were added followed by water, and then dichloromethane was added, the sample was centrifuged, and the bottom layer was collected and dried. Finally, ethyl acetate and water were added, the sample was centrifuged, and the supernatant was analysed by gas chromatography (Model CP3800, Varian Inc., Palo Alto, CA, USA).

A 2 × 3 factorial arrangement of treatments was applied. The factors were: (1) grain type, wheat, or corn; and (2) sNSP level, high (8.47 and 11.69 g/kg for starter, and 8.74 and 12.75 g/kg for grower, in corn and wheat diets, respectively), medium (6.22 and 9.95 g/kg for starter and 8.01 and 11.45 g/kg for grower, in corn and wheat diets, respectively) or low (5.78 and 9.31 g/kg for starter and 6.32 and 9.95 g/kg for grower, in corn and wheat diets, respectively). This resulted in 6 dietary treatments, 8 replicate pens per treatment. The sNSP levels differed between the wheat- and corn-based diets due to the differing NSP compositions between these two primary grains, but need to ensure both diets met the nutrient requirements of the birds. Thus, the three sNSP levels represent the relative change in sNSP content within each diet, as opposed to the absolute sNSP level. The ingredient composition of the diets is presented in Table 2 and Table 3. Table 4 and Table 5 present the analysed nutrient composition of the diets. Titanium dioxide (TiO_2_) was added at a rate of 0.5% as an inert marker for nutrient digestibility assessment.

### 2.3. Performance Data Collection

The body weight and feed intake of the birds were recorded on an individual pen basis on arrival and on bird age d 14 and d 35, for determination of individual feed intake (FI), body weight gain (BWG), and feed conversion ratio (FCR), corrected for mortality, at d 0–14, d 14–35 and d 0–35. The number of male birds per pen was determined, and used to calculate the percentage male birds per pen.

### 2.4. Sample Collection

#### 2.4.1. Excreta Collection

On d 14 and d 35, excreta samples were collected from each pen by placing the birds onto a clean metal tray and collecting the fresh excreta as soon as possible post-defecation. Samples were then homogenised, weighed, frozen at −20 °C, and then freeze-dried to constant weight. The freeze-dried samples were then ground and homogenized. Approximately 1.5 g of ground samples was weighed into a crucible, oven-dried at 105 °C to constant weight, and reweighed to determine the dry matter content.

#### 2.4.2. Ileal Digesta Collection

Four (d 14) and two (d 35) birds per pen were randomly selected, respectively, for ileum digesta sample collection. Birds were weighed individually before being euthanised by cervical dislocation. Ileum digesta samples were then pooled per pen, homogenised, frozen at −20 °C, and then freeze dried for further chemical analyses.

#### 2.4.3. Measurements and Chemical Analyses

The diet and freeze-dried excreta and ileum samples were ground through a 0.5 mm screen. Total dry matter content was determined in the diets and freeze-dried excreta and ileum samples by weighing approximately 1.5 g of sample into a crucible and drying it to constant weight in a 105 °C oven. Gross energy was determined in the diets, excreta, and ileum samples using an adiabatic bomb calorimeter (Model 6400, Parr Instruments, Moline, IL, USA), using benzoic acid as a calibration standard. Nitrogen content of these samples was determined using a combustion analyser (Leco model FP-2000N analyser, Leco Corp., St. Joseph, MI, USA) using EDTA as a calibration standard, and the value was multiplied by 6.25 to calculate the protein content. Total starch was measured using the Megazyme total starch assay (Megazyme International Ireland Ltd., Wicklow, Ireland). The calcium content of the diets was measured using the microwave digestion technique (Milestone UltraWAVE, Milestone Srl, Sorisole (BG), Italy) and the total phosphorus concentration was determined using an inductively coupled plasma optical emission spectrometer (ICP-OES, Agilent, Melbourne, Australia). The NSP content and composition of the diets, excreta, and ileum samples was analysed by gas-liquid chromatography, as described above, and titanium dioxide (TiO_2_) content was quantified using UV-spectroscopy, according to the method described by Short et al. [32].

### 2.5. Calculation of Nutrient Digestibility and Flow of NSP Sugar Residues

The following equation was used for calculation of NSP degradability at the total tract level and for calculation of apparent ileal or total tract digestibility of nutrients and metabolisability of crude protein and gross energy:

Digestibility (%) = 1 − [1 × (TiO_2_,_% diet_/TiO_2_,_% digesta/excreta_) × (nutrient_% digesta/excreta_/nutrient_% diet_)] × 100,

Nutrient refers to either dry matter, gross energy, crude protein, starch (assuming all glucose measured were derived from starch), or NSP.

Total tract flow of NSP sugar residues was calculated as g/kg DM intake using the equation:

NSP sugar residue flow _excreta_ = NSP_excreta_ × (TiO_2diet_/TiO_2excreta_)

### 2.6. Statistical Analysis

All data were analysed using Minitab (Minitab 19. 2020.1, Minitab Inc., State College, PA, USA). Data were tested for normality using the Anderson–-Darling test, and then analysed as a 2 × 3 factorial arrangement using General Linear Model (GLM). Percentage of males was used as a co-variate when analyzing the performance data, after first checking the model assumption. Tukey’s multiple range test was used to determine the differences between individual treatment means when interactions were observed. Pen served as the experimental unit, and there were eight replicate pens per treatment. Significant differences between mean values were declared at *p* < 0.05.

## 3. Results

### 3.1. Growth Performance

Growth performance at d 0–14, 14–35 and 0–35 are presented in Table 6. At d 0–14, birds fed the corn-based diet presented higher body weight gain and feed intake (*p* = 0.003 and 0.034, respectively), and showed a tendency for improved FCR (*p* = 0.058), compared to those fed the wheat-based diets. Furthermore, at this age BWG was greater in birds fed the low sNSP level compared to those fed the medium sNSP level (*p* = 0.014). At d 14–35, birds fed the diets with medium sNSP level presented greater FI compared to those fed the low sNSP level (*p* = 0.026), but sNSP level had no impact on BWG or FCR, and grain source had no impact on any of the performance parameters measured in this period. Over the whole trial period (d 0–35), birds fed the corn-based diet presented a lower FCR (*p* = 0.049), and tendency for greater BWG (*p* = 0.053), compared to those fed the wheat-based diets. Dietary sNSP level had no impact on d 0–35 performance. No interactions between grain type and sNSP level were observed on productive performance in this study.

### 3.2. Nutrient Utilisation

The impact of the dietary treatments on nutrient utilisation, measured at ileal and total tract level, at both d 14 and d 35, are presented in Table 7 and Table 8, respectively. As illustrated in Table 7, birds fed the corn-based diet presented higher apparent ileal digestibility of energy (*p* = 0.010) and total tract digestibility of DM (*p* < 0.001), starch (*p* < 0.001), and apparent total tract protein (*p* = 0.001) and energy (*p* < 0.001) metabolism at d 14 compared to those fed the wheat-based diet. However, apparent ileal protein digestibility was greater in birds fed wheat compared to those fed corn (*p* < 0.001). Apparent ileal starch digestibility at d 14 was greater in birds fed the high sNSP level compared to those fed the medium or low sNSP level (*p* = 0.010). Similarly, apparent total tract starch digestibility at d 14 was greater in birds fed the high sNSP level compared to those fed the low sNSP level (*p* = 0.019).

Table 8 presents that at d 35, birds fed the corn-based diets presented higher apparent ileal energy (*p* = 0.015) and starch (*p* = 0.003) digestibility and higher apparent total tract DM (*p* = 0.028), and starch (*p* < 0.001) digestibility and gross energy metabolisability (*p* = 0.007) compared to those fed the wheat-based diets. Birds fed the medium sNSP level also presented higher total tract DM (*p* = 0.001) and energy (*p* = 0.031) digestibility at d 35 compared to those fed the high or low sNSP levels. No interactions between grain type and iNSP level were observed for any of the measurements, at both bird ages.

### 3.3. Utilisation of NSP and Their Constituent Sugars

Table 9 presents the influence of the dietary treatments on the apparent total tract disappearance of soluble, insoluble, total NSP and free oligosaccharides (FO), as well as the ratio of soluble to insoluble NSP (sNSP:iNSP) in the excreta, at both d 14 and d 35. At d 35, an interaction between grain source and sNSP was observed for apparent total tract sNSP degradability, showing that when the medium sNSP level was fed, sNSP degradation was greater in birds fed the corn-based diet compared to those fed the wheat-based diet (*p* = 0.034). Degradability of sNSP at d 14 (*p* < 0.001), total NSP at d 14 (*p* = 0.017), and digestibility of FO at d 35 (*p* = 0.001) was higher in birds fed corn compared to those fed wheat. At d 14, birds fed the diets with high sNSP level presented greater apparent total tract sNSP degradability (*p* = 0.030), and lower apparent insoluble NSP degradation (*p* = 0.021), compared to those fed the diets with low sNSP level. At both d 14 and d 35, the excreta sNSP:iNSP ratio was lower in birds fed corn compared to those fed wheat (*p* < 0.001 for both), and at d 14 this ratio value was higher in birds fed the high sNSP level compared to the medium or low sNSP level (*p* = 0.014).

Table 10 presents the apparent total tract flow of the constituent soluble and insoluble sugars at d 14. Birds fed the wheat-based diets presented greater apparent total tract flow of insoluble ribose (*p* < 0.001), arabinose (*p* = 0.006), xylose (*p* < 0.001), mannose (*p* = 0.036) and glucose (*p* < 0.001), and soluble arabinose (*p* < 0.001), xylose (*p* < 0.001), galactose (*p* = 0.016), and glucose (*p* < 0.001), but lower insoluble galactose flow (*p* < 0.001) compared to those fed the corn-based diets. Soluble arabinose and glucose flow was greater in birds fed the high sNSP level compared to those fed the medium or low sNSP level (*p* = 0.013 and *p* < 0.001, respectively). The opposite was true for insoluble galactose and fucose flow, with greater levels seen in birds fed the low sNSP level compared to those fed the medium or high sNSP level (*p* = 0.011 and *p* = 0 001, respectively). Apparent insoluble mannose and soluble xylose flow were greater in birds fed the high sNSP level compared to those fed the low sNSP level (*p* = 0.029 and *p* = 0.026, respectively). No interactions between grain type and sNSP level were observed for any measurements of total tract flow of NSP sugar residue at d 14.

Table 11 shows the impact of the dietary treatments on apparent soluble constituent sugar flow at d 35. An interaction between grain source and sNSP level was observed for apparent soluble rhamnose total tract flow, showing that feeding the medium sNSP level increased the apparent flow of total tract soluble rhamnose in corn-fed birds compared to feeding the low or high sNSP level (*p* = 0.040), but in wheat-fed birds. Birds fed the wheat-based diets presented greater apparent total tract flow of soluble fucose (*p* = 0.001), arabinose (*p* < 0.001), xylose (*p* < 0.001), and glucose (*p* < 0.001) compared to those fed the corn-based diets. Apparent total tract flow of soluble glucose was greater in birds fed the high sNSP level compared to those fed the low sNSP level (*p* = 0.012).

Table 12 illustrates the impact of the dietary treatments on apparent insoluble constituent sugar flow at d 35. An interaction between grain source and sNSP level was observed for apparent flow of insoluble xylose, showing that flow of insoluble xylose was greater in birds fed the high sNSP level compared to the low sNSP level, but only when the wheat-based diets were fed (*p* = 0.004). Birds fed the corn-based diet presented higher apparent total tract flow of rhamnose (*p* < 0.001), fucose (*p* < 0.001), arabinose (*p* = 0.002) and galactose (*p* < 0.001), and lower apparent flow of mannose (*p* = 0.026), compared to those fed the wheat-based diets. Apparent insoluble glucose, mannose, and ribose flow was greater in birds fed the diets with medium sNSP level compared to those fed the high or low sNSP level (*p* < 0.001 for all). Similarly, apparent insoluble rhamnose flow was greater in birds fed the medium sNSP level diets compared to those fed the high sNSP level (*p* = 0.008). In contrast, apparent insoluble arabinose flow was greater in birds fed the diets with high sNSP level compared to those fed the medium sNSP level (*p* = 0.019).

## 4. Discussion

It is well-established that dietary NSP regulates digestion and modulates gastrointestinal health in poultry, directly influencing bird performance and litter quality [33,34]. Traditionally, only the anti-nutritive effects of sNSP have been considered, but a recent report has highlighted the positive impact of sNSP on broiler gastrointestinal health and performance, namely through its role as a source of substrates for probiotic bacteria species [35]. In the current study, the extent to which sNSP level and composition influences bird performance and nutrient utilisation in common commercial wheat and corn-based diets was investigated. Although significant responses to sNSP were observed, it must be noted that formulating the diets to keep crude fibre, protein, and energy levels similar meant that the three sNSP levels were not extremely different from each other within each grain source. Thus, further research is warranted using more extreme differences in sNSP to determine the optimum sNSP level to formulate to, to derive more meaningful performance data. Removing the constraint of crude fibre values during feed formulation would enable for a greater range of sNSP levels. The contribution of the other grain sources on performance also warrants consideration; regression analysis including the grain sources revealed that barley had a positive impact on growth performance at d 14, without considering the impact of its sNSP content.

As expected, performance was better in birds fed the corn-based diets, based on d 0–35 cFCR, compared to those fed the wheat-based diets, which is in agreement with a number of studies [36,37,38]. This is likely because the sNSP content of the wheat-based diets was approximately 50% higher than that in the corn-based diets, owing to much lower levels of sNSP in corn. The corn used in this study contained around 0.3% sNSP, whilst the wheat contained approximately 1.4% sNSP, which is indeed much lower than the values reported previously by Back Knudsen [13], who indicated that the sNSP content ranged from 0.2–1.6% and 1.9–2.9% (DM basis) for corn and wheat, respectively. The wheat-based diet also contained a higher concentration of high molecular weight sNSP, such as arabinoxylans [39,40]; the estimated soluble arabinoxylan content of the corn- and wheat-based diets in the current study were approximately 0.20% and 0.54%, respectively. The presence of these polymers results in increased viscosity in the gastrointestinal tract [41], inducing a barrier between enzymes and perspective substrates, and between nutrients and the gastrointestinal lining. The consequence of this could impair nutrient utilisation, resulting in increasing undigested nutrient presence in the gastrointestinal tract. This, in turn, can alter the microbiota profile by providing fuel for pathogenic bacterial species, which then compete with the host for nutrients [18,42]. This may partly explain why birds fed the wheat-based diet, which had higher total protein content in the starter phase, presented lower performance. It is interesting to note that BWG was higher, and FI was numerically highest, in birds fed the low sNSP level compared to the medium sNSP level at d 0–14. This could again be related to viscosity, whereby sNSP induces signals of satiety due to prolonged digesta transit rates and heightened water retention in the gastrointestinal tract, suggesting birds fed the low sNSP diets in the current study probably had comparatively lower feeling of satiation and thus could consume more feed. However, this heightened feed intake was coupled with increased weight gain, suggesting that the NSP in this diet was at a low enough level to not induce anti-nutritional effects on viscosity. At d 35, total tract DM and metabolisability of gross energy was greater in birds fed the medium sNSP level compared to the high or low sNSP level, possibly indicating that the sNSP adequately stimulated the microbiota environment towards proficient manufacture of SCFA [41], providing energy. However, this did not translate into improvements in performance, suggesting that nutrient partitioning between the host and the microbiota was not improved with this treatment, likely due to an alteration in the intestinal microbial profile [43]. Additionally, the wheat-based diets with medium sNSP level contained the highest free oligosaccharide level, which may have fuelled bacteria earlier in the gastrointestinal tract. This might have caused undesirable proliferation of bacterial population in the upper gut, potentially resulting in nutrient competition with the host, as opposed to the desirable fermentation in caeca which is the main site for bacterial fermentation.

Results from this study suggest that considering the quantity of sNSP alone during feed formulation does not provide an accurate indication of the impact of dietary NSP on gastrointestinal status and bird performance, due to the variable ability of the constituent sugars to modulate microbiota composition, and influence of the sNSP on digesta viscosity. There are a lack of studies examining NSP content in commercial-type diets, with most studies investigating effects of fibre addition in purified or semi-purified diets. Thus, further research is needed into the influence of the composition and physiochemical properties of dietary fibre, as opposed to the total level, in common diets, with particular focus on the quantity of fermentable and non-fermentable fractions, and ratio between the soluble and insoluble components in the sNSP [13]. This was illustrated by Musigwa et al. [26], who observed that the proportion of total NSP that was soluble, as opposed to total level, directly influenced the efficacy of a multi-carbohydrase at enhancing energy utilisation in broilers. For this reason, it is likely that further refinements in the definition of sNSP to take into account sugar composition, molecular weight, and degree of substitution will further benefit the predictability of bird performance and response to exogenously added enzymes.

The lower insoluble NSP digestibility observed at d 14 in birds fed the high sNSP level, compared to the low sNSP level, may be a consequence of young birds possessing microbiota that is not yet adapted to digesting complex, insoluble NSP molecules. The suggestion is that the microbiota will preferentially ferment sNSP over iNSP, and as a result, overwhelming a naïve microbiota with sNSP can compromise its ability, and indeed need, to ferment insoluble NSP. However, exposure to these dietary polymers, or ideally oligosaccharides derived from them, in young birds can prime the microbiota to become more adept at utilising more complex NSP later in life [44]. This is elucidated by the significantly greater impact of sNSP level on the flow of soluble constituent sugars at d 14 than at d 35, and conversely, on the flow of the insoluble constituent sugars at d 35 than d 14. Regardless of the initial sNSP:iNSP ratio in diets, the ratio of soluble to insoluble NSP in the excreta was lowest in diets with the medium and low sNSP levels at d 14, and tended to be lower with the low sNSP level at d 35, revealing a more efficient breakdown of sNSP in birds fed the low sNSP diets. This reconfirms that young birds have a requirement for a minimum sNSP level in order to optimise early sNSP fermentation, which then influences the fermentation of total NSP at a later growing stage. This could also partially be explained by the observed greater sNSP degradability at d 35 in birds fed the corn-based diet with medium or low sNSP, compared to those fed the wheat-based diet with medium sNSP level. This inherent requirement for dietary fibre has also been demonstrated previously by Sadeghi et al. [45], in that broilers actively selected extra fibre sources to boost their immunity, regardless of impact on performance. Thus, it appears that it is beneficial to supplement diets containing poor fermentable NSP substrates, such as corn, with a fermentable NSP substrate, to enhance intestinal fermentation activities. Arabinoxylans (AX) are the predominant NSP in both wheat and corn, but the structure of AX in corn is more complex than that in wheat, with a higher degree of substitution and arabinose: xylose ratio [46], meaning corn AX is less readily fermentable than wheat AX [47]. In addition, the poor ileal and total tract starch digestibility observed at d 14 in birds fed the low sNSP diets could be attributed to production of high molecular weight viscous AX, from insoluble NSP degradation, exceeding their hydrolysis rate in the gastrointestinal tract in this treatment group. The NSP digestibility values in this study were lower than those presented by Jamroz et al. [48] and Zhang et al. [49], but higher than those seen by Meng et al. [50], probably due to differences in diet composition, such as source of fat and quality of wheat, and bird age.

## 5. Conclusions

In conclusion, outputs from this study indicated that considering sNSP level in commercial-type broiler diets provides a more meaningful indication of bird response to dietary fibre, in terms of performance and nutrient utilisation, compared to using conventional crude fibre values. Outputs from this study also demonstrated a need to supply birds with fermentable fibre substrates, indicating that the industry should consider supplementing sNSP-poor diets with a source of fermentable NSP. In conclusion, the current results suggest that it may be advantageous to include sNSP levels when formulating broiler diets, although further research is required to define what levels to formulate to, depending on the composition of the NSP in feed ingredients.

## Figures and Tables

**Table 1:** Content of dry matter (g/100 g, as is basis) and non-starch polysaccharides of the plant-based ingredients used in the study diets (g/kg, as is basis).

Ingredients	DM ^1^	sNSP ^2^	iNSP ^3^	tNSP ^4^
Wheat	90.53	14.23	83.08	97.31
Corn	88.97	2.86	64.56	67.42
Barley	90.96	42.36	137.4	179.7
Sorghum	88.45	1.65	53.54	55.2
Soybean meal	90.05	11.22	132.2	143.4
Canola meal	92.16	15.35	146.8	162.1
Wheat bran	91.21	23.16	385.1	408.2
Oat bran	92.40	52.24	65.02	117.3
Soy protein concentrate	93.05	14.69	157.4	172.1

^1^ Dry matter; ^2^ Soluble non-starch polysaccharides; ^3^ Insoluble non-starch polysaccharides; ^4^ Total non-starch polysaccharides.

**Table 2:** Composition of starter diets (g/100 g, as fed basis).

Ingredient	Corn-Based Diet			Wheat-Based Diet		
	High sNSP	Medium sNSP	Low sNSP	High sNSP	Medium sNSP	Low sNSP
Corn	51.25	52.00	51.40	-	-	-
Wheat	-	-	-	52.14	50.02	50.00
Sorghum	-	2.34	8.00	0.10	5.00	8.00
Barley	8.00	5.57	-	8.00	5.13	-
Wheat bran	0.13	2.24	2.73	1.54	2.03	2.96
Oat bran	3.47	-	-	-	-	-
Soybean meal	19.26	21.03	23.89	19.46	20.15	27.66
Soy protein concentrate	6.00	6.00	6.00	6.00	6.00	3.14
Canola meal solvent	5.00	4.00	1.39	5.00	4.00	0.01
Canola oil	2.39	2.35	2.09	3.47	3.31	3.63
Limestone	1.08	1.12	1.14	1.23	1.20	1.30
Dicalcium phosphate 18P/21Ca	1.69	1.63	1.67	1.36	1.45	1.50
Salt	0.32	0.32	0.33	0.30	0.30	0.37
TiO_2_	0.50	0.50	0.50	0.50	0.50	0.50
Vitamin Premix ^1^	0.050	0.050	0.050	0.050	0.050	0.050
Mineral Premix ^2^	0.075	0.075	0.075	0.075	0.075	0.075
Choline	0.12	0.12	0.12	0.10	0.10	0.10
L-Lysine HCl	0.24	0.23	0.30	0.22	0.22	0.21
DL-Methionine	0.30	0.31	0.32	0.31	0.32	0.35
L-Threonine	0.10	0.10	0.09	0.13	0.13	0.13
Calculated Values (as-is)						
Crude fibre, %	3.30	3.30	3.20	3.50	3.50	3.48
AME_n_, kcal/kg	3008	3008	3008	3008	3008	3008
d Arg, %	1.280	1.279	1.305	1.315	1.307	1.316
d Lys, %	1.180	1.180	1.180	1.180	1.180	1.180
d Met, %	0.597	0.604	0.614	0.581	0.589	0.608
d Met + Cys, %	0.880	0.880	0.880	0.880	0.880	0.880
d Ileu, %	0.785	0.771	0.770	0.709	0.758	0.719
d Threo, %	0.690	0.690	0.690	0.690	0.690	0.690
d Val, %	0.906	0.893	0.890	0.800	0.846	0.800
Fat, %	5.38	5.32	4.83	5.87	5.67	5.58
Calcium, %	0.90	0.90	0.90	0.90	0.90	0.93
Available P, %	0.45	0.45	0.45	0.45	0.46	0.46

^1^ Vitamin premix per kg diet: vitamin A, 12 MIU; vitamin D, 5 MIU; vitamin E, 75 mg; vitamin K, 3 mg; nicotinic acid, 55 mg; pantothenic acid, 13 mg; folic acid, 2 mg; riboflavin, 8 mg; cyanocobalamin, 0.016 mg; biotin, 0.25 mg; pyridoxine, 5 mg; thiamine, 3 mg; antioxidant, 50 mg; ^2^ Trace mineral concentrate supplied per kg diet: Cu (sulfate), 16 mg; Fe (sulfate), 40 mg; I (iodide), 1.25 mg; Se (selenate), 0.3 mg; Mn (sulfate and oxide), 120 mg; Zn (sulfate and oxide), 100 mg; cereal-based carrier, 128 mg; mineral oil, 3.75 mg.

**Table 3:** Composition of the grower diets (g/100 g, as fed basis).

Ingredient	Corn-Based Diet			Wheat-Based Diet		
	High sNSP	Medium sNSP	Low sNSP	High sNSP	Medium sNSP	Low sNSP
Corn	49.72	54.62	58.28	-	-	-
Wheat	-	-	-	59.65	57.08	55.01
Sorghum	-	-	-	-	4.00	7.83
Barley	8.00	4.03	-	8.00	3.34	-
Wheat bran	1.93	2.82	3.60	-	-	-
Oat bran	2.00	1.03	-	-	-	
Soybean meal	18.76	18.25	19.82	14.11	16.68	22.68
Soy Protein Concentrate	6.00	6.00	5.17	6.00	6.00	0.53
Canola meal solvent	6.00	6.00	6.00	4.79	5.51	6.00
Canola oil	3.80	3.41	3.28	3.51	3.61	4.03
Limestone	1.04	1.05	1.06	1.17	1.15	1.15
Dicalcium phosphate 18P/21Ca	1.47	1.46	1.44	1.25	1.25	1.27
Salt	0.32	0.32	0.33	0.30	0.30	0.30
TiO_2_	0.50	0.50	0.50	0.50	0.50	0.50
Vitamin Premix ^1^	0.050	0.050	0.050	0.050	0.050	0.050
Mineral Premix ^2^	0.075	0.075	0.075	0.075	0.075	0.075
L-lysine HCl	0.08	0.11	0.11	0.21	0.13	0.199
DL-Methionine	0.22	0.23	0.23	0.26	0.24	0.27
L-Threonine	0.03	0.04	0.04	0.11	0.08	0.11
Calculated Values (as-is)						
Crude fibre, %	3.60	3.60	3.60	3.00	3.00	3.00
AME_n_, kcal/kg	3086	3086	3086	3086	3086	3086
d Arg, %	1.263	1.232	1.224	1.172	1.245	1.171
d Lys, %	1.050	1.050	1.050	1.050	1.050	1.050
d Met, %	0.518	0.523	0.525	0.512	0.504	0.517
d Met + Cys, %	0.800	0.800	0.800	0.800	0.800	0.800
d Ileu, %	0.785	0.771	0.770	0.709	0.758	0.719
d Threo, %	0.690	0.690	0.690	0.690	0.690	0.690
d Val, %	0.906	0.893	0.890	0.800	0.846	0.800
Fat, %	6.90	6.59	6.51	5.91	6.11	6.64
Calcium, %	0.84	0.84	0.84	0.84	0.84	0.84
Available P, %	0.42	0.42	0.42	0.42	0.42	0.42

^1^ Vitamin premix per kg diet: vitamin A, 12 MIU; vitamin D, 5 MIU; vitamin E, 75 mg; vitamin K, 3 mg; nicotinic acid, 55 mg; pantothenic acid, 13 mg; folic acid, 2 mg; riboflavin, 8 mg; cyanocobalamin, 0.016 mg; biotin, 0.25 mg; pyridoxine, 5 mg; thiamine, 3 mg; antioxidant, 50 mg; ^2^ Trace mineral concentrate supplied per kg diet: Cu (sulfate), 16 mg; Fe (sulfate), 40 mg; I (iodide), 1.25 mg; Se (selenate), 0.3 mg; Mn (sulfate and oxide), 120 mg; Zn (sulfate and oxide), 100 mg; cereal-based carrier, 128 mg; mineral oil, 3.75 mg.

**Table 4:** Analysed nutrient composition of starter diets (as-fed basis).

Item	Corn-Based Diet			Wheat-Based Diet		
	High sNSP	Medium sNSP	Low sNSP	High sNSP	Medium sNSP	Low sNSP
DM, %	88.67	87.19	87.74	88.57	88.43	88.28
Gross energy, kcal/kg	16.77	16.45	16.50	16.82	16.75	16.68
Crude fibre, %	2.45	2.40	2.40	2.51	2.50	2.57
Crude protein, %	20.93	21.36	21.91	23.18	23.40	23.46
Starch, %	38.06	37.73	37.45	36.45	35.03	35.24
Calcium, %	1.04	0.99	0.96	1.00	1.01	1.03
Total phosphorus, %	0.72	0.72	0.66	0.75	0.74	0.75
Total NSP, g/kg	75.31	76.25	76.43	84.73	85.69	83.83
Soluble NSP, g/kg	8.47	6.22	5.78	11.69	9.95	9.32
Rhamnose	0.04	0.05	0.04	0.05	0.03	0.04
Fucose	0.07	0.06	0.07	0.06	0.06	0.06
Ribose	0.39	0.39	0.39	0.37	0.41	0.42
Arabinose	1.62	1.40	1.40	2.91	2.75	2.58
Xylose	0.72	0.66	0.73	2.82	2.65	2.73
Mannose	1.34	1.40	1.38	1.84	1.34	1.52
Galactose	1.87	1.77	1.74	2.28	2.19	2.18
Glucose	3.44	1.22	0.73	2.79	1.74	0.95
Insoluble NSP, g/kg	66.84	70.03	70.65	73.03	75.75	74.51
Rhamnose	0.59	0.60	0.62	0.57	0.57	0.61
Fucose	0.92	0.97	1.03	0.97	0.93	1.04
Ribose	0.27	0.24	0.26	0.27	0.25	0.24
Arabinose	16.99	17.90	18.37	20.56	20.46	20.82
Xylose	14.85	15.42	15.47	18.90	20.49	19.11
Mannose	2.05	2.06	2.05	1.64	2.08	1.87
Galactose	15.36	16.73	17.14	13.75	14.37	15.49
Glucose	23.97	24.65	24.34	25.38	25.95	24.52
sNSP:iNSP	0.13	0.09	0.08	0.16	0.13	0.13
Free oligosaccharides, g/kg	34.76	34.04	35.93	36.22	37.50	40.08

**Table 5:** Analysed nutrient composition of grower diets (as-fed basis).

Item	Corn-Based Diet			Wheat-Based Diet		
	High sNSP	Medium sNSP	Low sNSP	High sNSP	Medium sNSP	Low sNSP
DM, %	90.92	87.62	89.72	90.21	89.90	90.58
Gross energy, kcal/kg	17.46	16.88	17.15	16.97	16.84	17.05
Crude fibre, %	2.50	2.63	2.48	2.74	2.82	2.97
Crude protein, %	21.61	20.81	21.04	20.99	20.71	21.14
Starch, %	36.44	36.04	36.32	39.15	39.91	37.03
Calcium, %	1.04	0.99	0.96	1.00	1.01	1.03
Total phosphorus, %	0.72	0.72	0.66	0.75	0.74	0.75
Total NSP, g/kg	80.14	84.10	83.56	87.45	79.62	78.63
Soluble NSP, g/kg	8.74	8.01	6.32	12.75	11.45	9.95
Rhamnose	0.05	0.05	0.05	0.05	0.04	0.03
Fucose	0.06	0.06	0.06	0.05	0.05	0.05
Ribose	0.42	0.41	0.40	0.40	0.39	0.41
Arabinose	1.71	1.65	1.55	3.38	3.09	3.18
Xylose	0.84	0.76	0.69	3.68	3.64	3.30
Mannose	1.61	1.78	1.62	1.63	1.57	1.28
Galactose	1.74	1.80	1.72	2.30	2.26	2.02
Glucose	3.34	2.46	0.99	2.84	1.84	0.94
Insoluble NSP, g/kg	71.40	76.09	77.23	74.69	68.17	68.67
Rhamnose	0.72	0.65	0.63	0.49	0.42	0.49
Fucose	1.01	1.01	1.00	0.81	0.77	0.85
Ribose	0.27	0.29	0.28	0.23	0.19	0.24
Arabinose	18.99	20.82	20.87	20.07	18.69	19.52
Xylose	16.31	18.95	18.20	21.63	19.12	17.94
Mannose	1.92	1.97	2.27	1.94	1.82	1.88
Galactose	15.72	16.08	16.77	11.96	12.11	12.34
Glucose	25.21	25.67	26.68	26.80	23.49	23.88
sNSP:iNSP	0.12	0.11	0.08	0.17	0.17	0.14
Free oligosaccharides, g/kg	36.81	35.32	34.04	35.35	42.70	36.03

**Table 6:** Effect of dietary soluble non-starch polysaccharide (sNSP) level and grain source on body weight gain (BWG, g/bird), feed intake (FI, g/bird), and feed conversion ratio corrected to mortality (cFCR) in broilers during d 0–14, 14–35 and 0–35.

Grain	sNSP Level	Day 0–14			Day 14–35			Day 0–35		
		BWG	FI	cFCR	BWG ^c^	FI	cFCR ^c^	BWG ^c^	FI	cFCR
Corn	High	482	583	1.227	1914	2950	1.547	2399	3533	1.478
	Medium	465	584	1.225	1966	3095	1.579	2432	3679	1.517
	Low	487	601	1.234	1912	2907	1.515	2396	3508	1.459
Wheat	High	448	569	1.271	1880	2934	1.570	2331	3504	1.510
	Medium	446	557	1.248	1910	3063	1.596	2352	3619	1.532
	Low	475	587	1.235	1881	2946	1.571	2358	3533	1.502
SEM		10	12	0.016	42	69	0.025	44	73	0.021
Grain										
Corn		478 ^a^	589 ^a^	1.229	1931	2984	1.547	2409	3573	1.485 ^b^
Wheat		457 ^b^	571 ^b^	1.251	1890	2981	1.579	2347	3552	1.515 ^a^
sNSP level										
High		465 ^ab^	576	1.249	1897	2942 ^ab^	1.558	2365	3518	1.494
Medium		456 ^b^	570	1.237	1938	3079 ^a^	1.587	2392	3649	1.524
Low		481 ^a^	594	1.235	1896	2927 ^b^	1.543	2377	3521	1.481
*p*-value										
Grain		0.003	0.034	0.058	0.180	0.953	0.081	0.053	0.682	0.049
sNSP level		0.014	0.068	0.568	0.431	0.026	0.137	0.783	0.071	0.063
Grain × sNSP level		0.411	0.756	0.325	0.933	0.821	0.644	0.855	0.795	0.755

Male percentage was used as covariate when analysing the d 35 performance data; ^a,b^ Means in the same column with no common superscripts are significantly different (*p* < 0.05). ^c^ Means in the same column were adjusted to 41.9% male birds as a covariate, if the covariate effect was significant.

**Table 7:** Effect of dietary soluble non-starch polysaccharide (sNSP) level and grain source on apparent ileal and total tract digestibility of dry matter (DM) and starch (%) and apparent total tract metabolisability of crude protein (CP) and gross energy (%) in broilers at d 14.

Items	Ileum				Total Tract			
	DM	CP	Energy	Starch	DM	CP	Energy	Starch
Grain								
Corn	66.62	81.34 ^b^	74.16 ^a^	97.71	73.26 ^a^	71.06 ^a^	75.78 ^a^	98.36 ^a^
Wheat	65.43	82.97 ^a^	72.58 ^b^	97.62	70.70 ^b^	66.18 ^b^	73.20 ^b^	97.81 ^b^
sNSP level								
High	66.27	81.77	73.33	98.16 ^a^	71.42	68.32	74.23	98.35 ^a^
Medium	65.91	82.44	73.60	97.49 ^b^	71.85	68.61	74.53	98.07 ^ab^
Low	65.89	82.25	73.17	97.34 ^b^	71.72	68.94	74.71	97.83 ^b^
SEM	0.94	0.55	0.83	0.31	0.72	1.84	0.81	0.20
*p*-value								
Grain	0.084	<0.001	0.010	0.700	<0.001	0.001	<0.001	<0.001
sNSP level	0.869	0.391	0.832	0.010	0.782	0.928	0.790	0.019
Grain × sNSP level	0.862	0.444	0.915	0.489	0.956	0.566	0.949	0.395

^a,b^ Means in the same column within main effects or interactive effect with no common superscripts are significantly different (*p* < 0.05).

**Table 8:** Effect of dietary soluble non-starch polysaccharide (sNSP) level and grain source on apparent ileal and total tract digestibility of dry matter (DM) and starch (%) and metabolisability of gross energy (%) and crude protein (%) in broilers at d 35.

Items	Ileum				Total Tract			
	DM	Protein	Energy	Starch	DM	Protein	Energy	Starch
Grain								
Corn	66.12	82.30	72.81 ^a^	96.74 ^a^	70.68 ^a^	61.07	75.42 ^a^	97.60 ^a^
Wheat	64.85	82.31	70.81 ^b^	94.50 ^b^	69.51 ^b^	59.58	73.79 ^b^	94.95 ^b^
sNSP level								
High	66.08	82.33	71.88	96.44	69.41 ^b^	60.65	74.06 ^b^	96.68
Medium	65.97	82.95	72.56	95.87	71.52 ^a^	61.12	75.73 ^a^	96.56
Low	64.41	81.63	70.99	94.56	69.36 ^b^	59.21	74.03 ^b^	95.59
SEM	1.17	0.61	1.10	1.01	0.73	1.78	0.81	0.80
*p*-value								
Grain	0.132	0.978	0.015	0.003	0.028	0.243	0.007	<0.001
sNSP level	0.194	0.059	0.267	0.100	0.001	0.443	0.031	0.251
Grain × sNSP level	0.953	0.834	0.656	0.294	0.925	0.519	0.748	0.220

^a,b^ Means in the same column within main effects or interactive effect with no common superscripts are significantly different (*p* < 0.05).

**Table 9:** Effect of dietary soluble non-starch polysaccharide (sNSP) level and grain source on apparent disappearance of soluble (sNSP), insoluble (iNSP), and total NSP (tNSP) and free oligosaccharides (FO) (%) and ratio of soluble to insoluble non-starch polysaccharide (sNSP:iNSP) in the excreta in broilers at d 14 and 35.

Grain	sNSP Level	Day 14					Day 35				
		sNSP	iNSP	tNSP	FO	sNSP:iNSP	sNSP	iNSP	tNSP	FO	sNSP:iNSP
Corn	High	35.18	13.13	15.61	98.73	0.09	41.72 ^ab^	14.56	18.07	98.83	0.07
	Medium	26.31	18.96	19.56	98.64	0.08	49.14 ^a^	22.30	24.78	99.06	0.07
	Low	21.95	20.54	20.65	98.66	0.08	43.09 ^a^	18.26	20.28	99.06	0.06
Wheat	High	13.25	13.10	13.12	98.69	0.16	29.14 ^ab^	19.05	20.66	98.70	0.16
	Medium	9.46	16.92	16.05	98.52	0.14	20.92 ^b^	14.64	15.73	98.80	0.15
	Low	5.08	16.03	15.17	98.42	0.14	23.29 ^ab^	21.42	21.71	98.32	0.14
SEM		4.42	2.30	2.17	0.13	0.01	6.72	3.61	3.77	0.15	0.01
Grain											
Corn		27.81 ^a^	17.54	18.60 ^a^	98.68	0.09 ^b^	44.65	18.37	21.04	98.98 ^a^	0.07 ^b^
Wheat		9.27 ^b^	15.35	14.78 ^b^	98.54	0.15 ^a^	24.56	18.37	19.37	98.61 ^b^	0.15 ^a^
sNSP level											
High		24.22 ^a^	13.11 ^b^	14.36	98.71	0.13 ^a^	35.60	16.80	19.37	98.77	0.12
Medium		17.89 ^ab^	17.94 ^ab^	17.80	98.58	0.11 ^b^	35.03	18.47	20.25	98.93	0.11
Low		13.51 ^b^	18.28 ^a^	17.91	98.54	0.11 ^b^	33.19	19.84	21.00	98.69	0.10
*p*-value											
Grain		<0.001	0.184	0.017	0.144	<0.001	0.002	0.999	0.533	0.001	<0.001
sNSP level		0.030	0.021	0.113	0.313	0.014	0.902	0.626	0.883	0.190	0.060
Grain × sNSP level		0.751	0.535	0.725	0.690	0.735	0.034	0.115	0.158	0.070	0.997

^a,b^ Means in the same column within main effects or interactive effect with no common superscripts are significantly different (*p* < 0.05).

**Table 10:** Effect of dietary soluble NSP (sNSP) level and grain source on apparent total tract flow (g/kg DM intake) of soluble and insoluble non-starch polysaccharide sugar residues at d 14.

Items		Grain		sNSP Level			SEM	*p*-Values		
		Corn	Wheat	High	Medium	Low		Grain	sNSP Level	Grain × sNSP Level
Rhamnose	Soluble	0.06	0.07	0.06	0.07	0.07	0.01	0.124	0.287	0.106
	Insoluble	0.68	0.59	0.60	0.63	0.68	0.13	0.335	0.795	0.794
Fucose	Soluble	0.39	0.42	0.40	0.42	0.40	0.03	0.183	0.778	0.263
	Insoluble	1.24	1.20	1.19 ^b^	1.19 ^b^	1.28 ^a^	0.03	0.086	0.001	0.360
Ribose	Soluble	0.07	0.08	0.07	0.07	0.08	0.01	0.150	0.824	0.411
	Insoluble	0.12 ^b^	0.17 ^a^	0.14	0.15	0.14	0.01	<0.001	0.419	0.356
Arabinose	Soluble	1.22 ^b^	2.65 ^a^	2.14 ^a^	1.83 ^b^	1.83 ^b^	0.13	<0.001	0.013	0.294
	Insoluble	21.40 ^b^	22.71 ^a^	21.76	21.75	22.65	0.64	0.006	0.190	0.556
Xylose	Soluble	0.75 ^b^	3.71 ^a^	2.44 ^a^	2.22 ^ab^	2.04 ^b^	0.17	<0.001	0.026	0.456
	Insoluble	21.74 ^b^	25.62 ^a^	24.12	23.66	23.25	0.65	<0.001	0.309	0.987
Mannose	Soluble	0.60	0.69	0.64	0.64	0.65	0.07	0.104	0.968	0.995
	Insoluble	1.76 ^b^	1.95 ^a^	1.98 ^a^	1.90 ^ab^	1.68 ^b^	0.12	0.036	0.029	0.283
Galactose	Soluble	2.26 ^b^	2.54 ^a^	2.44	2.34	2.40	0.16	0.016	0.758	0.674
	Insoluble	17.82 ^a^	16.01 ^b^	16.44 ^b^	16.65 ^b^	17.66 ^a^	0.47	<0.001	0.011	0.310
Glucose	Soluble	0.84 ^b^	1.62 ^a^	1.71 ^a^	1.09 ^b^	0.89 ^b^	0.13	<0.001	< 0.001	0.257
	Insoluble	8.52 ^b^	11.95 ^a^	11.06	10.83	8.81	1.04	<0.001	0.303	0.571

^a,b^ Means in the same row within main effects or interactive effect with no common superscripts are significantly different (*p* < 0.05).

**Table 11:** Effect of dietary soluble NSP (sNSP) level and grain source on apparent total tract flow (g/kg DM intake) of soluble non-starch polysaccharide sugar residues at d 35.

Grain	sNSP Level	Rhamnose	Fucose	Ribose	Arabinose	Xylose	Mannose	Galactose	Glucose
	High	0.04 ^b^	0.26	0.09	1.29	0.82	0.51	1.40	1.06
Corn	Medium	0.10 ^a^	0.21	0.10	1.09	0.68	0.62	1.73	0.69
	Low	0.06 ^b^	0.21	0.11	1.04	0.61	0.58	1.49	0.58
	High	0.05 ^b^	0.33	0.12	2.78	4.00	0.46	1.71	1.68
Wheat	Medium	0.06 ^ab^	0.28	0.11	2.53	3.97	0.52	2.01	1.63
	Low	0.05 ^b^	0.29	0.12	2.50	3.41	0.53	1.58	0.96
SEM		0.01	0.03	0.02	0.25	0.38	0.07	0.18	0.22
Grain									
Corn		0.07	0.23 ^b^	0.10	1.14 ^b^	0.70 ^b^	0.57	1.54	0.78 ^b^
Wheat		0.06	0.30 ^a^	0.12	2.61 ^a^	3.79 ^a^	0.50	1.77	1.42 ^a^
sNSP									
High		0.05	0.30	0.10	2.03	2.41	0.49	1.56	1.37 ^a^
Medium		0.08	0.24	0.11	1.81	2.32	0.57	1.87	1.16 ^ab^
Low		0.06	0.25	0.11	1.77	2.01	0.55	1.54	0.77 ^b^
*p*-value									
Grain		0.060	0.001	0.127	<0.001	<0.001	0.191	0.074	<0.001
sNSP level		<0.001	0.066	0.765	0.422	0.433	0.317	0.061	0.012
Grain × sNSP level		0.040	0.961	0.559	0.991	0.730	0.874	0.743	0.374

^a,b^ Means in the same column within a main effect or interactive effect with no common superscripts are significantly different (*p* < 0.05).

**Table 12:** Effect of dietary soluble NSP (sNSP) level and grain source on apparent total tract flow (g/kg DM intake) of insoluble non-starch polysaccharide sugar residues at d 35.

Grain	sNSP Level	Rhamnose	Fucose	Ribose	Arabinose	Xylose	Mannose	Galactose	Glucose
	High	0.76	1.20	0.13	24.87	25.86 ^ab^	1.07	17.47	4.31
Corn	Medium	0.97	1.18	0.18	22.39	23.34 ^b^	1.54	16.25	10.15
	Low	0.82	1.28	0.15	25.97	26.20 ^ab^	1.11	17.76	5.12
	High	0.59	0.99	0.15	23.39	28.73 ^a^	1.37	13.43	6.99
Wheat	Medium	0.66	1.04	0.20	20.92	25.02 ^ab^	1.71	13.58	9.83
	Low	0.60	1.05	0.14	21.54	22.38 ^b^	1.27	13.66	5.88
SEM		0.05	0.04	0.012	1.05	1.15	0.129	0.72	1.05
Grain									
Corn		0.85 ^a^	1.22 ^a^	0.15	24.13 ^a^	25.14	1.24 ^b^	17.16 ^a^	6.53
Wheat		0.62 ^b^	1.03 ^b^	0.16	21.95 ^b^	25.38	1.45 ^a^	13.56 ^b^	7.57
sNSP									
High		0.68 ^b^	1.10	0.14 ^b^	24.13 ^a^	27.30	1.22 ^b^	15.45	5.65 ^b^
Medium		0.81 ^a^	1.11	0.19 ^a^	21.65 ^b^	24.18	1.62 ^a^	14.92	9.99 ^a^
Low		0.71 ^ab^	1.17	0.14 ^b^	23.76 ^ab^	24.29	1.19 ^b^	15.71	5.50 ^b^
*p*-value									
Grain		<0.001	<0.001	0.350	0.002	0.768	0.026	<0.001	0.185
sNSP level		0.008	0.118	<0.001	0.019	0.004	<0.001	0.438	<0.001
Grain × sNSP level		0.282	0.388	0.236	0.184	0.004	0.781	0.444	0.293

^a,b^ Means in the same column within a main effect or interactive effect with no common superscripts are significantly different (*p* < 0.05).

## Data Availability

All available data are incorporated in the manuscript.

## References

[1] Choct M. (2015). Feed non-starch polysaccharides for monogastric animals: Classification and function. Anim. Prod. Sci..

[2] Bedford M.R. (1996). Interaction between ingested feed and the digestive system in poultry. J. Appl. Poult. Res.

[3] Iji P.A. (1999). The impact of cereal non-starch polysaccharides on intestinal development and function in broiler chickens. Worlds Poult. Sci. J..

[4] Kheravii S.K., Morgan N.K., Swick R.A., Choct M., Wu S.-B. (2018). Roles of dietary fibre and ingredient particle size in broiler nutrition. Worlds Poult. Sci. J..

[5] Choct M. (2009). Managing gut health through nutrition. Br. Poult. Sci..

[6] Mahmood T., Guo Y. (2020). Dietary fiber and chicken microbiome interaction: Where will it lead to?. Anim. Nutr..

[7] Branton S.L., Lott B.D., Deaton J.W., Maslin W.R., Austin F.W., Pote L.M., Keirs R.W., Latour M.A., Day E.J. (1997). The effect of added complex carbohydrates or added dietary fiber on necrotic enteritis lesions in broiler chickens. Poult. Sci..

[8] Adibmoradi M., Navidshad B., Jahromi M.F. (2016). The effect of moderate levels of finely ground insoluble fibre on small intestine morphology, nutrient digestibility and performance of broiler chickens. Ital. J. Anim. Sci..

[9] Bach Knudsen K.E. (2001). The nutritional significance of “dietary fibre” analysis. Anim. Feed Sci. Tech..

[10] Theander O., Åman P., Westerlund E., Graham H. (1994). Enzymatic/chemical analysis of dietary fiber. J. AOAC Int..

[11] Jha R., Fouhse J.M., Tiwari U.P., Li L., Willing B.P. (2019). Dietary Fiber and Intestinal Health of Monogastric Animals. Front. Vet. Sci..

[12] Raninen K., Lappi J., Mykkänen H., Poutanen K. (2019). Dietary fiber type reflects physiological functionality: Comparison of grain fiber, inulin, and polydextrose. Nut. Rev..

[13] Jiménez-Moreno E., Frikha M., de Coca-Sinova D., García J., Mateos G.G. (2013). Oat hulls and sugar beet pulp in diets for broilers 1. Effects on growth performance and nutrient digestibility. Anim. Feed Sci. Tech..

[14] Bach Knudsen K.E. (1997). Carbohydrate and lignin contents of plant materials used in animal feeding. Anim. Feed Sci. Tech..

[15] Morgan N.K., Choct M., Toghyani M., Wu S.-B. Effects of dietary insoluble and soluble non-starch polysaccharides on performance and ieal and exreta moisture contents in broilers. Proceedings of the 29th Annual Australian Poultry Science Symposium.

[16] Owusu-Asiedu A., Patience J.F., Laarveld B., Van Kessel A.G., Simmins P.H., Zijlstra R.T. (2006). Effects of guar gum and cellulose on digesta passage rate, ileal microbial populations, energy and protein digestibility, and performance of grower pigs. J. Anim. Sci..

[17] Konieczka P., Smulikowska S. (2018). Viscosity negatively affects the nutritional value of blue lupin seeds for broilers. Animal.

[18] Bedford M.R., Cowieson A.J. (2012). Exogenous enzymes and their effects on intestinal microbiology. Anim. Feed Sci. Tech..

[19] Bao Y.M., Choct M. (2010). Dietary NSP nutrition and intestinal immune system for broiler chickens. Worlds Poult. Sci. J..

[20] Rodehutscord M., Rückert C., Maurer H.P., Schenkel H., Schipprack W., Bach Knudsen K.E., Schollenberger M., Laux M., Eklund M., Siegert W. (2016). Variation in chemical composition and physical characteristics of cereal grains from different genotypes. Arch. Anim. Nutr..

[21] Choct M. (1997). Feed non-starch polysaccharides: Chemical structures and nutritional significance. Feed Milling Int..

[22] Kiarie E., Romero L.F., Ravindran V. (2014). Growth performance, nutrient utilization, and digesta characteristics in broiler chickens fed corn or wheat diets without or with supplemental xylanase. Poult. Sci..

[23] Munyaka P.M., Nandha N.K., Kiarie E., Nyachoti C.M., Khafipour E. (2016). Impact of combined β-glucanase and xylanase enzymes on growth performance, nutrients utilization and gut microbiota in broiler chickens fed corn or wheat-based diets. Poult. Sci..

[24] Saleh A.A., Kirrella A.A., Abdo S.E., Mousa M.M., Badwi N.A., Ebeid T.A., Nada A.L., Mohamed M.A. (2019). Effects of dietary xylanase and arabinofuranosidase combination on the growth performance, lipid peroxidation, blood constituents, and immune response of broilers fed low-energy diets. Animals.

[25] Gracia M.I., Aranibar M.J., Lazaro R., Medel P., Mateos G.G. (2003). Alpha-amylase supplementation of broiler diets based on corn. Poult. Sci..

[26] Musigwa S., Cozannet P., Morgan N., Swick R.A., Wu S.-B. (2020). Multi-carbohydrase effects on energy utilization depend on soluble non-starch polysaccharides-to-total non-starch polysaccharides in broiler diets. Poult. Sci..

[27] Maharjan P., Mayorga M., Hilton K., Weil J., Beitia A., Caldas J., England J., Coon C. (2019). Non-cellulosic polysaccharide content in feed ingredients and ileal and total tract non-cellulosic polysaccharide digestibility in 21-and 42-day-old broilers fed diets with and without added composite enzymes. Poult. Sci..

[28] Cobb-Vantress Cobb 500 Broiler: Performance and Nutrition Supplement, 14, Cobb-Vantress.com. https://www.cobb-vantress.com/assets/Cobb-Files/product-guides/bdc20a5443/70dec630-0abf-11e9-9c88-c51e407c53ab.pdf.

[29] Englyst H.N., Quigley M.E., Hudson G.J. (1994). Determination of dietary fibre as non-starch polysaccharides with gas–liquid chromatographic, high-performance liquid chromatographic or spectrophotometric measurement of constituent sugars. Analyst.

[30] Theander O., Åman P., Westerlund E., Andersson R., Pettersson D. (1995). Total dietary fiber determined as neutral sugar residues, uronic acid residues, and Klason lignin (the Uppsala method): Collaborative study. J. AOAC Int..

[31] Morgan N.K., Keerqin C., Wallace A., Wu S.-B., Choct M. (2019). Effect of arabinoxylo-oligosaccharides and arabinoxylans on net energy and nutrient utilization in broilers. Anim. Nutr..

[32] Short F.J., Gorton P., Wiseman J., Boorman K.N. (1996). Determination of titanium dioxide added as an inert marker in chicken digestibility studies. Anim. Feed Sci. Tech..

[33] Williams P.E.V., Geraert P.A., Uzu G., Annison G., Morand-Fehr P. (1997). Factors affecting non-starch polysaccharide digestibility in poultry. Feed Manufactoring in Southern Europe: New Challages.

[34] Ward N.E. (2020). Debranching enzymes in corn/soybean meal-based poultry feeds: A review. Poult. Sci..

[35] Singh A.K., Kim W.K. (2021). Effects of dietary fiber on nutrients utilization and gut health of poultry: A review of challenges and opportunities. Animals.

[36] Rodríguez M.L., Rebolé A., Velasco S., Ortiz L.T., Treviño J., Alzueta C. (2012). Wheat-and barley-based diets with or without additives influence broiler chicken performance, nutrient digestibility and intestinal microflora. J. Sci. Food Agric..

[37] Yaghobfar A., Kalantar M. (2017). Effect of Non-Starch Polysaccharide (NSP) of Wheat and Barley Supplemented with Exogenous Enzyme Blend on Growth Performance, Gut Microbial, Pancreatic Enzyme Activities, Expression of Glucose Transporter (SGLT1) and Mucin Producer (MUC2) Genes of Broiler Chickens. Braz. J. Poult. Sci..

[38] Ghiasvand A.R., Khatibjoo A., Mohammadi Y., Gharaei M.A., Shirzadi H. (2021). Effect of fennel essential oil on performance, serum biochemistry, immunity, ileum morphology and microbial population, and meat quality of broiler chickens fed corn or wheat-based diet. Br. Poult. Sci..

[39] Annison G., Choct M. (1991). Anti-nutritive activities of cereal non-starch polysaccharides in broiler diets and strategies minimizing their effects. Worlds Poult. Sci. J..

[40] Bedford M.R., Schulze H. (1998). Exogenous enzymes for pigs and poultry. Nutr. Res. Rev..

[41] Nguyen H.T., Bedford M.R., Wu S.-B., Morgan N.K. (2021). Soluble non-starch polysaccharide modulates broiler gastrointestinal tract environment. Poult. Sci..

[42] Saki A.A., Matin H.R.H., Tabatabai M.M., Zamani P., Harsini R.N. (2010). Microflora population, intestinal condition and performance of broilers in response to various rates of pectin and cellulose in the diet. Eur. Poult. Sci..

[43] Rinttilä T., Apajalahti J. (2013). Intestinal microbiota and metabolites—Implications for broiler chicken health and performance1. J. Appl. Poult. Res..

[44] Bautil A., Verspreet J., Buyse J., Goos P., Bedford M.R., Courtin C.M. (2019). Age-related arabinoxylan hydrolysis and fermentation in the gastrointestinal tract of broilers fed wheat-based diets. Poult. Sci..

[45] Sadeghi A., Toghyani M., Gheisari A. (2015). Effect of various fiber types and choice feeding of fiber on performance, gut development, humoral immunity, and fiber preference in broiler chicks. Poult. Sci..

[46] Vangsøe C.T., Bonnin E., Joseph-Aime M., Saulnier L., Neugnot-Roux V., Bach Knudsen K.E. (2021). Improving the digestibility of cereal fractions of wheat, maize, and rice by a carbohydrase complex rich in xylanases and arabinofuranosidases: An in vitro digestion study. J. Sci. Food Agric..

[47] Malunga L.N., Beta T. (2016). Isolation and identification of feruloylated arabinoxylan mono-and oligosaccharides from undigested and digested maize and wheat. Heliyon.

[48] Jamroz D., Jakobsen K., Bach Knudsen K.E., Wiliczkiewicz A., Orda J. (2002). Digestibility and energy value of non-starch polysaccharides in young chickens, ducks and geese, fed diets containing high amounts of barley. Comp. Biochem. Physiol. Part A Mol. Integr. Physiol..

[49] Zhang L., Xu J., Lei L., Jiang Y., Gao F., Zhou G.H. (2014). Effects of xylanase supplementation on growth performance, nutrient digestibility and non-starch polysaccharide degradation in different sections of the gastrointestinal tract of broilers fed wheat-based diets. Asian-Australas. J. Anim. Sci..

[50] Meng X., Slominski B.A., Nyachoti C.M., Campbell L.D., Guenter W. (2005). Degradation of cell wall polysaccharides by combinations of carbohydrase enzymes and their effect on nutrient utilization and broiler chicken performance. Poult. Sci..

